# Microbial community assembly and pathogen signatures in groundwater and tap water systems in greater Cairo, Egypt

**DOI:** 10.1186/s42506-026-00211-8

**Published:** 2026-03-19

**Authors:** Neveen M. Rizk, Ayda K. Kelany, Sayeda M. Abdo, Mohammed Yosri, Fagr Kh. Abdel-Gawad, Khaled Haider, Akram B. Sultan, Ahmed M. Younis, Mahmoud Gad

**Affiliations:** 1 Water Pollution Research Department, National Research Centre, Giza, 12622 Egypt; 2 Department of Genomic Medicine, Cairo University Hospitals, Cairo University, Cairo, 11511 Egypt; 3 Applied Science Research Center, Applied Science Private University, Amman, 11831 Jordan; 4 MEU Research Unit, Middle East University, Amman, 11831 Jordan; 5 Hydrobiology Laboratory, Water Pollution Research Department, National Research Centre, Giza, 12622 Egypt; 6 The Regional Center for Mycology and Biotechnology, Al-Azhar University, Cairo, 11787 Egypt; 7 Center of Excellence for Research and Applied Studies on Climate Change and Sustainable Development (C3SD-NRC), National Research Centre, Dokki, Giza, 12622 Egypt; 8 Department of Medical Laboratory Sciences, College of Applied Medical Sciences, University of Bisha, Bisha, Saudi Arabia; 9 Department of Biology, Faculty of Science, University of Bisha, Bisha, Saudi Arabia; 10 Botany and Microbiology Department, Faculty of Science, Al-Azhar University, Cairo, Egypt

**Keywords:** 16S rRNA amplicon sequencing, Prokaryotic communities, Waterborne pathogens, Ecosystem dynamics, Environmental factors, Groundwater, Tap water

## Abstract

**Background:**

Microbial communities in aquatic ecosystems are integral to water quality and public health, yet their structure and underlying ecological processes in regions like Egypt remain underexplored. To address this gap, this research explores the structure and dynamics of prokaryotic communities in tap water and groundwater in Cairo, Egypt.

**Methods:**

Using environmental DNA metabarcoding, bioinformatics, and statistical modeling, we investigated microbial composition, pathogen identification, environmental factors, and ecological assembly processes.

**Results:**

The sequence analysis revealed the presence of 6,868 amplicon sequence variants (ASVs), with distinct community structures between groundwater and tap water. Proteobacteria dominated both habitats, with significant habitat-specific variations in Firmicutes, Actinobacteria, Verrucomicrobia, and Bacteroidota. Key genera included *Methylobacterium* in tap water, and *Thauera* and *Legionella* in groundwater, reflecting habitat-specific adaptations. The potential presence of *Legionella*—detected through 16 S rRNA gene signatures—may indicate conditions that could support organisms associated with diseases such as Legionnaires’ disease; however, 16 S-based detection does not confirm viability or infectivity. Similarly, the surrogate presence of taxa such as *Streptococcus salivarius*, *Stenotrophomonas maltophilia*, and *Acinetobacter baumannii* in tap-water samples suggests possible post-treatment contamination or biofilm-associated persistence, warranting further targeted monitoring using methods capable of confirming viability. Ecological assessments indicated that stochastic mechanisms, particularly ecological drift, were the dominant forces shaping microbial community assembly in both water sources, whereas homogeneous selection exerted a moderate influence specifically within groundwater environments. Environmental parameters such as DO%, NO₂-N, and NO₃-N were critical in shaping tap water communities, while NH₄-N and TDS influenced groundwater communities.

**Conclusion:**

This study highlights the distinct microbial dynamics of groundwater and tap water, emphasizing the importance of integrated water quality management strategies to mitigate nutrient pollution, monitor potential pathogen signatures, and protect public health.

## Introduction

 Microbial communities are essential to aquatic ecosystems, significantly impacting water quality, nutrient cycling, and the stability of the overall ecosystem. In drinking water systems, these communities are directly linked to public health and safety, as they can harbor pathogenic species or facilitate beneficial microbial processes [1, 2]. Although microbial communities play a crucial role, their dynamics in tap water and groundwater systems remain poorly understood in developing countries like Egypt. These systems are critical for supporting human populations, making it essential to investigate their microbial composition, ecological processes, and the environmental factors shaping them [3].

Egypt’s drinking water treatment and distribution systems face several region-specific challenges. Aging infrastructure contributes to significant water loss (reported to reach ~ 35% of total drinking-water production in the distribution systems) and can impede the maintenance of adequate disinfectant residuals [4, 5]. In some areas, poorly maintained distribution networks allow secondary contamination of water [6], as intruding pollutants and biofilm growth can reintroduce microbes after treatment. Chlorine disinfection is the primary treatment method nationwide, but chlorine residuals often decay over long pipeline distances, necessitating booster rechlorination stations to sustain microbial control [6, 7]. These infrastructure and treatment gaps have occasionally manifested in public health incidents [4, 8], underscoring the need for improved network management and monitoring in Egypt’s water systems.

Microbial community structure is shaped by various environmental parameters, such as temperature, pH, dissolved oxygen (DO), and nutrients like nitrogen and phosphorus [9]. Tap water systems, often influenced by treatment processes, exhibit distinct microbial profiles compared to natural groundwater [10]. Treatment methods, such as chlorination, can alter microbial diversity and select for specific taxa, while groundwater microbial communities typically reflect natural, untreated conditions. Recent advances in environmental DNA metabarcoding have enabled detailed analyses of microbial diversity and ecological interactions, offering a powerful tool to explore these complex systems [11].

The presence of waterborne pathogens remains a major public health concern, especially in areas with insufficient water treatment and sanitation infrastructure. Contaminated drinking water can serve as a reservoir for bacteria, viruses, and protozoa, leading to the transmission of a wide range of diseases. The World Health Organization has emphasized that limited access to safe water and adequate sanitation infrastructure heightens the vulnerability of populations to otherwise preventable diseases [12]. Among bacterial pathogens, *Streptococcus salivarius*, though commonly regarded as an oral commensal, has been implicated in opportunistic infections, including endocarditis, meningitis, and septicemia in immunocompromised individuals, underscoring its clinical relevance under certain conditions [13, 14]. Similarly, the Gram-negative and non-fermentative bacterium *Stenotrophomonas maltophilia*, ubiquitous in aquatic environments and hospital water systems, is increasingly recognized as an opportunistic pathogen. Its intrinsic resistance to multiple antibiotics and association with pneumonia, bloodstream infections, and urinary tract infections make it a growing concern in immunocompromised populations [15, 16]. Comparing groundwater and tap water is particularly important in Egypt because each source entails distinct contamination pathways. Untreated shallow wells used in rural areas are vulnerable to fecal contamination from sewage and agricultural runoff, and studies from the Nile Delta have reported fecal indicator bacteria in well water exceeding safety limits [17]. By contrast, municipal tap water is centrally treated, yet microbial regrowth and re-contamination can occur during distribution and household storage due to biofilms in aging pipes and tanks, which may harbor opportunistic pathogens (e.g., *Legionella*, *Pseudomonas*) that tolerate disinfection [18]. The detection of such pathogens in drinking water supplies highlights potential inefficiencies in disinfection and underscores the importance of continuous microbiological surveillance and advanced treatment technologies.

From a microbial-ecological perspective, it is important to distinguish between deterministic (niche‐based) and stochastic processes that govern community assembly in water systems [19]. Deterministic processes reflect consistent ecological “filters”—including nutrient availability, disinfectant exposure, pH/redox conditions, and other physicochemical constraints—that impose directional selection and favor taxa with traits adapted to local conditions [20, 21]. In contrast, stochastic processes, such as dispersal and ecological drift, represent probabilistic components of assembly driven by random colonization and demographic fluctuations, yielding community variation shaped by chance and historical contingency [21, 22]. Differentiating between ecological drift and selection is more than theoretical – it directly informs water quality management strategies. If neutral processes like drift dominate in a drinking water habitat, microbial community composition may shift unpredictably even under stable environmental conditions, implying that managers should minimize random events and maintain system stability. Conversely, if communities are shaped largely by selection, controlling key environmental parameters (such as residual chlorine, nutrient concentrations, or pipe materials) could steer the microbiome toward a safer composition [23–25]. Recent research in Egypt’s aquatic environments indeed suggests that stochastic drift can be a dominant force in community assembly [10]. Recognizing whether random drift or deterministic selection prevails in groundwater and tap water microbiomes helps water authorities devise appropriate interventions – from adjusting treatment protocols to managing distribution infrastructure – to ensure a resilient and healthy drinking water supply.

Despite the pivotal role of microbial communities in drinking water supply systems, studies examining their composition and ecological behavior, particularly in developing countries like Egypt, remain limited. This study investigates prokaryotic assemblages in aquatic systems (i.e., groundwater and tap water), focusing on their structural composition, the key environmental factors shaping their distribution, the ecological mechanisms influencing their community formation, and the occurrence of potential pathogens. By employing environmental DNA metabarcoding alongside advanced bioinformatics and robust statistical modeling, this research offers a valuable understanding of pathogen occurrence, microbial structural patterns, assembly dynamics, and associated environmental influences. These outcomes carry important implications for monitoring microbial water quality and protecting public health, especially in water-scarce regions where sustainable resource management is essential.

## Methods

### Study area and sample collection

A total of ten water samples were obtained from Al-Kalyubia, located in the Greater Cairo region of Egypt, encompassing two distinct water sources: groundwater (*n* = 4), obtained from 4 wells used directly for drinking and not subjected to any drinking-water treatment processes, and tap water (*n* = 6), representing treated municipal drinking water from household distribution outlets. Sampling sites were selected using a purposive strategy to capture the two principal drinking-water sources in the study area (directly consumed well water versus municipally treated tap water). Sampling was performed during the winter of 2022. For each site, approximately 1,000 mL of water was collected and transported on ice to the laboratory to ensure the preservation of microbial DNA. Upon arrival, the samples were filtered through 0.22 μm membrane filters (Millipore, Bedford, MA, USA) to concentrate microbial biomass for downstream DNA extraction.

### Physicochemical analysis

The physicochemical characteristics of the water samples were evaluated in accordance with the protocols outlined in the Standard Methods for the Examination of Water and Wastewater [26]. In situ measurements of temperature, dissolved oxygen (DO), and DO saturation were conducted using an AD 360 DO meter (Adwa Instruments, Inc., Europe). pH measurements were carried out using a Jenway 3510 bench-top meter, whereas total dissolved solids (TDS) and electrical conductivity (EC) were determined with a Jenway 4510 conductivity meter. Nitrogen-related parameters were analyzed using standard methods: Total Kjeldahl Nitrogen (TKN) was quantified through digestion with mercuric sulfate followed by titration, in accordance with method 4500-Norg. Ammonium (NH₄-N) concentrations were measured via ion chromatography using the ICS-5000 system. Nitrite (NO₂-N) was assessed through a colorimetric procedure (method 4500-B), and nitrate (NO₃-N) was quantified using a modified version of the sodium salicylate method [27]. The total nitrogen (TN) value was obtained by summing TKN, NO₂-N, NO₃-N, and NH₄-N. Total phosphorus (TP) levels were determined using the ascorbic acid method (method 4500-P).

### DNA extraction and 16 S rRNA gene sequencing

Genomic DNA was extracted from each water sample using the DNeasy PowerLyzer PowerSoil Kit (QIAGEN, USA) in accordance with the manufacturer’s guidelines. DNA concentrations were measured using the Qubit 2.0 fluorometer in combination with the Qubit dsDNA Broad Range Assay Kit (Life Technologies, Grand Island, NY, USA). For the amplification of prokaryotic communities, the V4–V5 region of the 16 S rRNA gene was targeted using the universal primers 515 F (5′-GTG YCA GCM GCC GCGGTA-3′) and 907R (5′-CCG YCA ATT YMT TTR AGTTT-3′), following the protocol of Quince et al. (2011). Each 25 µL PCR mixture contained 2.5 µL of 10× TransStart FastPfu buffer, 2.5 mM of dNTPs, 0.4 µM of each primer, 0.5 µL of TransStart FastPfu DNA polymerase (TransGen Biotech, China), 5 µg of bovine serum albumin (Sigma, Steinheim, Germany), 20 ng of extracted DNA, and nuclease-free water to adjust the final volume. To ensure reproducibility, triplicate PCR reactions were performed for each sample. The amplification conditions included an initial denaturation at 95 °C for 5 min, followed by 25 cycles of denaturation (95 °C for 30 s), annealing (55 °C for 30 s), and extension (72 °C for 90 s), with a final elongation step at 72 °C for 10 min. The resulting PCR products were purified and subjected to paired-end sequencing (2 × 300 bp) using the Illumina MiSeq platform (Illumina Inc., San Diego, CA, USA). Sequencing was carried out by Majorbio Bio-Pharm Technology Co., Ltd. (Shanghai, China). To ensure uniformity across samples, the sequencing dataset was rarefied to 91,000 reads per sample for subsequent analyses.

### Bioinformatics

The raw sequencing data were processed using the LotuS2 pipeline [28], a computationally efficient and accurate tool for processing microbial community data. Sequence quality control and denoising were carried out using the DADA2 ( RRID: SCR_023519) algorithm, which generated high-resolution amplicon sequence variants (ASVs) by correcting sequencing errors and distinguishing closely related taxa [28]. Taxonomic classification of the resulting ASVs was performed using the Ribosomal Database Project (RDP) classifier (RRID: SCR_022773) in combination with the SILVA (RRID: SCR_006423) reference database (version 138). A confidence threshold of 80% was applied to ensure reliable and consistent taxonomic assignments [29].

### Potential pathogens identification

Taxonomic identification of potential pathogens was conducted using BLAST version 2.14.0 (RRID: SCR_004870), applying stringent parameters (-evalue 1e-10, percent identity ≥ 97%, and query coverage per HSP ≥ 90%) against a curated pathogen reference database. This custom database included complete genome sequences of 876 known prokaryotic and eukaryotic pathogenic organisms [30]. The top (> 100 reads/aquatic habitat) human pathogens were only represented in this research. Importantly, 16 S rRNA–based detection of pathogen-associated taxa reflects surrogate presence and does not confirm viability, infectivity, or virulence; therefore, such findings should be interpreted cautiously and ideally complemented by targeted viability-aware methods.

### Analysis of microbial community assembly

To assess the ecological mechanisms driving the assembly of prokaryotic communities, the iCAMP framework (Integrated Community Assembly Mechanisms by Pairwise community similarity) was utilized. This method quantifies the contributions of deterministic and stochastic processes to community composition. Two principal metrics were applied: the weighted beta Net Relatedness Index (βNRI) and the modified Raup–Crick index (RC). Interpretation of the dominant ecological processes was based on the following thresholds: a βNRI value below − 1.96 was indicative of homogeneous selection (HoS), whereas a value above 1.96 reflected heterogeneous selection (HeS). When |βNRI| was less than or equal to 1.96, the RC value determined the stochastic process: RC > 0.95 indicated dispersal limitation (DL), RC < − 0.95 signified homogenizing dispersal (HD), and values of |RC| ≤ 0.95 suggested undominated processes, primarily ecological drift (DR) [22].

### Statistical analysis

Beta diversity patterns of prokaryotic communities were explored using non-metric multidimensional scaling (nMDS), based on Bray–Curtis dissimilarity matrices. To evaluate significant differences in community composition across different aquatic habitats, statistical analyses including analysis of similarity (ANOSIM) and permutational multivariate analysis of variance (Adonis) were applied. In addition, distance-based redundancy analysis (dbRDA) was conducted to investigate the relationships between microbial community structures and environmental variables, offering insights into how physicochemical conditions shape microbial assemblages [20].

## Results

### Structural patterns of prokaryotic communities in water systems

**Fig. 1:**
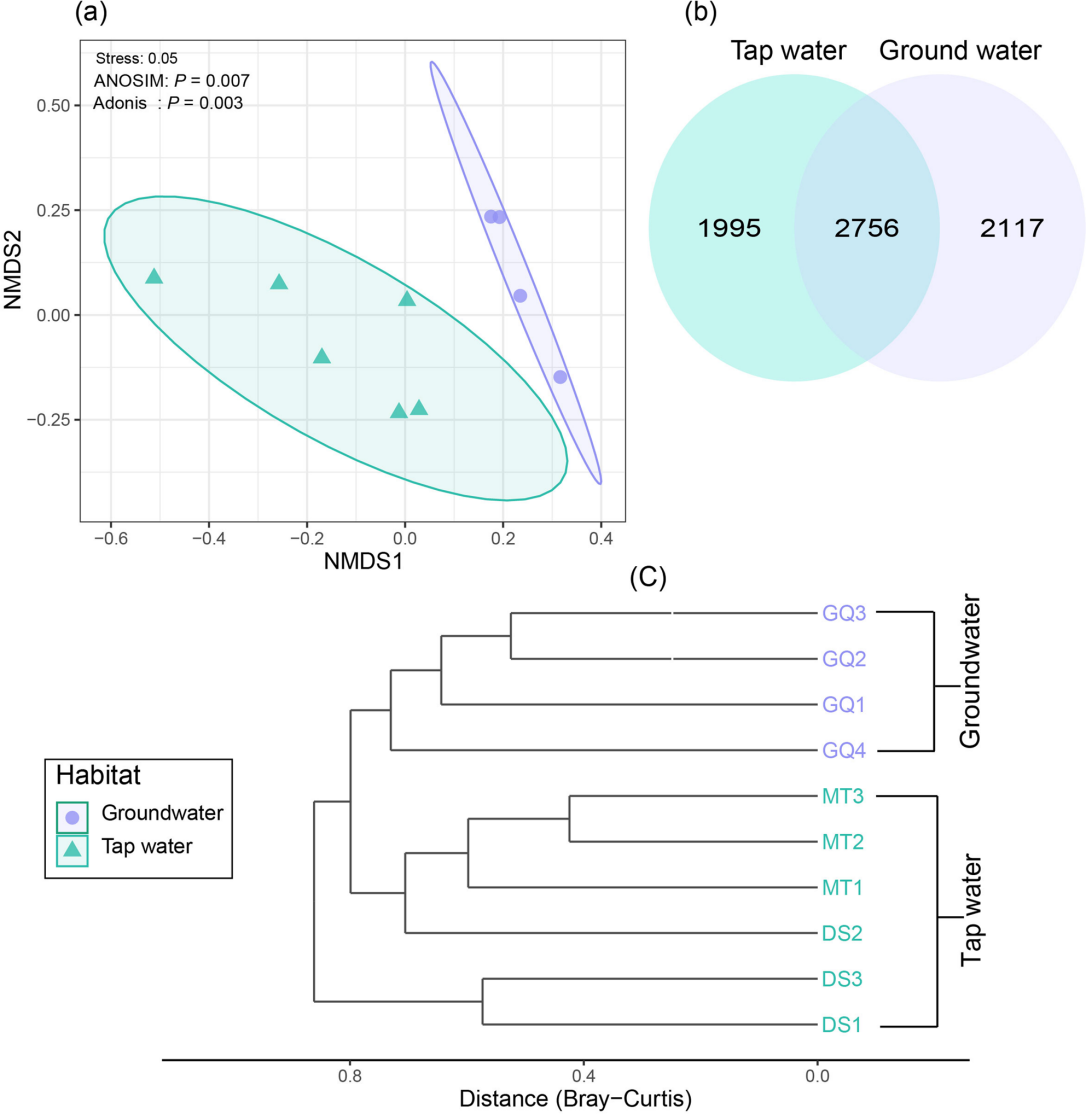
**a** PCoA plot of prokaryotic community composition in groundwater and tap water samples based on Bray-Curtis distances. **b** Venn diagram showing the prokaryotic species richness in groundwater and tap water samples. **c **Cluster analysis dendrogram of microbial community composition in groundwater (GQ1-GQ4) and tap water (DS1-DS3, MT1-MT3) samples based on Bray-Curtis distances

The sequencing process produced between 91,139 and 126,995 reads per sample. To ensure a consistent and unbiased analysis, the dataset was rarefied to a depth of 91,000 reads, leading to the identification of 6,868 ASVs. NMDS analysis, with a stress value of 0.05, effectively revealed distinct microbial compositions between groundwater and tap water, highlighting significant ecological variations between these two environments (Fig. 1a). The distinct clustering patterns observed in the NMDS plot suggest that microbial community compositions were influenced by environmental factors and water treatment processes. This differentiation was statistically validated using ANOSIM (*P* = 0.007) and Adonis (*P* = 0.003), further confirming the significance of these differences. A Venn diagram provided additional insights, revealing 2,117 unique species in groundwater, 1,995 unique species in tap water, and 2,756 species shared between the two habitats (Fig. 1b). These results were corroborated by cluster analysis based on Bray-Curtis distances, which emphasized habitat-specific microbial community structures (Fig. 1c).

### Taxonomic profiling of prokaryotic communities

**Fig. 2:**
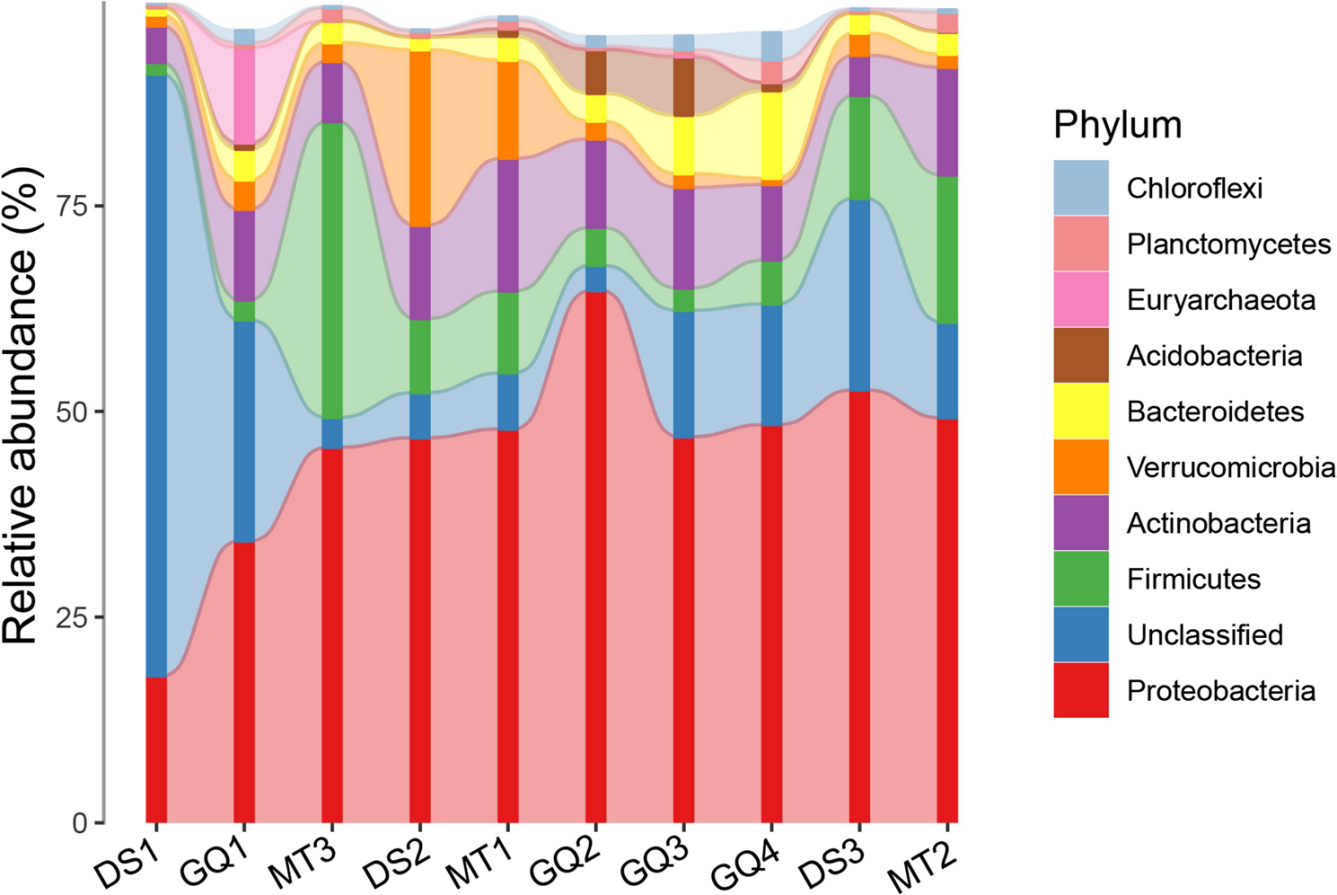
Relative abundance of top 10 phyla of prokaryotes in groundwater (GQ1-GQ4) and tap water (DS1-DS3, MT1-MT3) samples

The relative abundance of prokaryotic phyla showed significant differences between tap water and groundwater, highlighting habitat-specific microbial community dynamics. Proteobacteria dominated both habitats, with a higher relative abundance in groundwater (48.57%) compared to tap water (43.34%). Firmicutes were more prevalent in tap water (14.46%) than in groundwater (3.78%), likely due to their resilience in treated water conditions. Conversely, Actinobacteria exhibited a slightly higher abundance in groundwater (10.79%) than in tap water (9.53%). Verrucomicrobia was more abundant in tap water (6.86%) compared to groundwater (2.04%), whereas Bacteroidota showed the opposite trend, with a greater prevalence in groundwater (6.21%) than in tap water (2.18%) (Fig. 2).

**Fig. 3:**
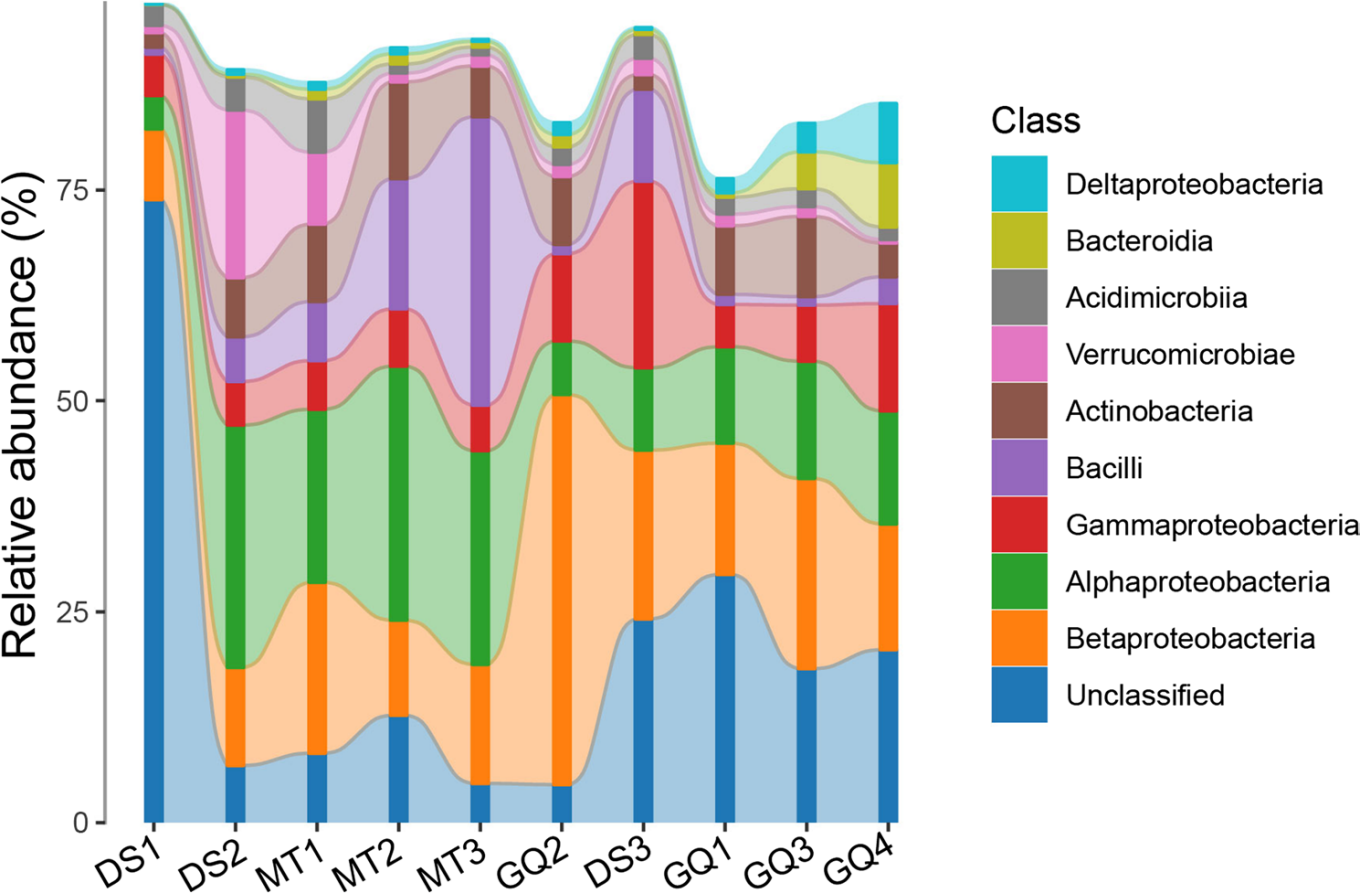
Relative abundance of top 10 classes of prokaryotes in groundwater (GQ1-GQ4) and tap water (DS1-DS3, MT1-MT3) samples

**Fig. 4:**
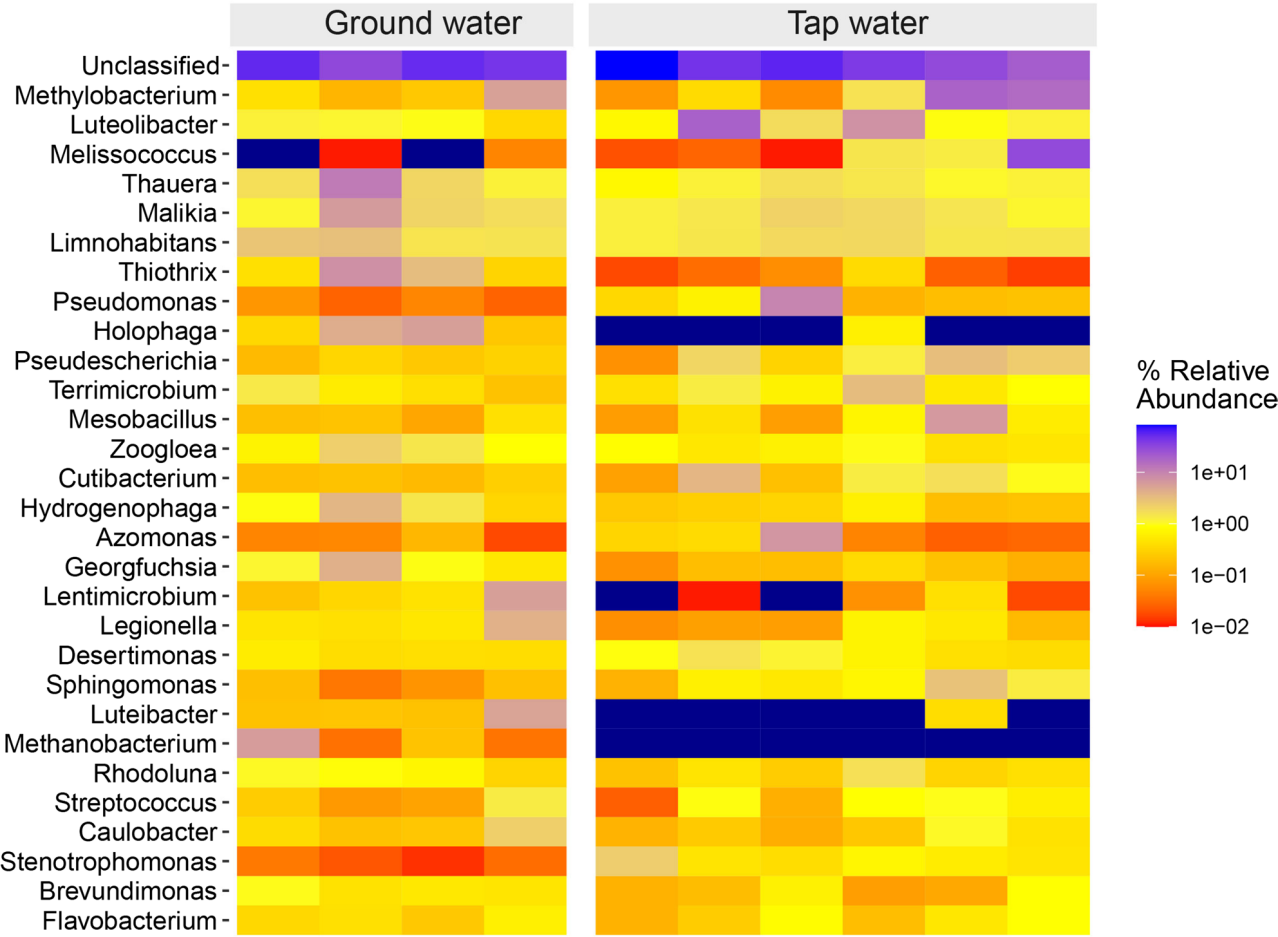
Relative abundance of top 30 genera of prokaryotes in groundwater and tap water samples

Less abundant phyla, such as Acidobacteria and Euryarchaeota, exhibited significantly higher relative abundances in groundwater (3.55% and 3.03%, respectively) than in tap water (0.19% and 0.009%). Betaproteobacteria were notably more abundant in groundwater (24.80%) compared to tap water (14.28%), suggesting their adaptation to natural aquifer conditions. Conversely, Alphaproteobacteria exhibited greater relative abundance in tap water (19.72%) than in groundwater (11.26%). Gammaproteobacteria showed similar distributions across both habitats, with a slight increase in groundwater (8.69%) compared to tap water (8.36%). Bacilli, however, were substantially more abundant in tap water (12.29%) than in groundwater (1.63%). Actinobacteria were slightly more prevalent in groundwater (7.43%) than in tap water (6.19%). Verrucomicrobiae were more abundant in tap water (5.60%) than in groundwater (1.10%). In contrast, Bacteroidia and Deltaproteobacteria were predominantly found in groundwater, with relative abundances of 3.50% and 3.38%, respectively, compared to 0.67% and 0.44% in tap water (Fig. 3). The relative abundance of prokaryotic genera exhibited clear habitat-specific differences between tap water and groundwater, revealing significant implications for water quality and public health. *Methylobacterium*, predominantly found in tap water (6.30%) compared to groundwater (1.62%) (Fig. 4).

Conversely, *Thauera* (4.16%) and *Malikia* (2.81%) were more prevalent in groundwater. Other noteworthy genera included *Holophaga* (2.69%) in groundwater, linked to organic-rich anaerobic environments, and *Thiothrix* (2.96%). In tap water, *Cutibacterium* (1.32%) was prominent. *Hydrogenophaga* was more abundant in groundwater (1.62%).

### Potential pathogens distribution in aquatic systems

**Fig. 5:**
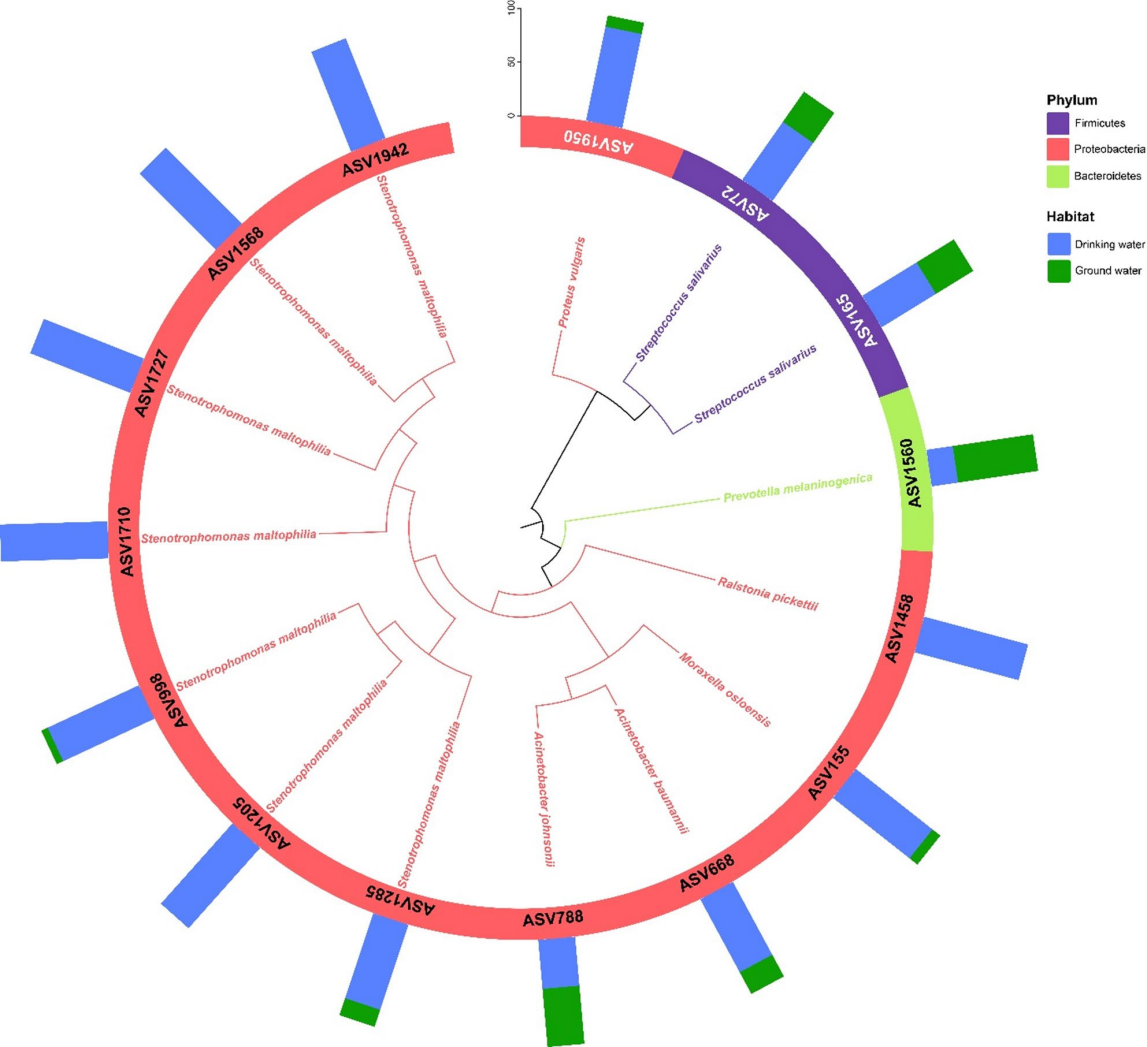
Top 15 potential human 16 S rRNA pathogens in aquatic habitat

This study identified the top 15 potential human pathogenic bacteria, highlighting the associated public health risks of waterborne microorganisms. Among these, *Streptococcus salivarius* (ASV72, ASV165) was the most abundant, with 4,059 and 395 total reads, respectively. Notably, 65.8% of ASV72 and 59.24% of ASV165 were traced to drinking water sources (Fig. 5). *Stenotrophomonas maltophilia*, a Gram-negative, non-fermentative bacterium, is increasingly recognized as both an environmental and nosocomial pathogen with global health implications. In this study, it was identified through multiple ASVs (ASV1285, ASV1205, ASV998, ASV1710, ASV1727, ASV1568, and ASV1942), several of which (e.g., ASV1205, ASV1710, ASV1727, ASV1568, ASV1942) were found in high relative abundance in drinking water samples (Fig. 5).

Several other clinically significant pathogens, including *Proteus vulgaris* (ASV1950), *Moraxella osloensis* (ASV155), and *Acinetobacter baumannii* (ASV668), exhibited high relative abundance (> 77.13%) in drinking water (Fig. 5). Of particular concern was the detection of *Acinetobacter johnsonii* (ASV788, 54.29%). *Ralstonia pickettii* (ASV1458) was detected exclusively in drinking-water samples. In contrast, *Prevotella melaninogenica* (ASV1560) was predominantly associated with groundwater, accounting for 75.25% of its total reads.

### The association between prokaryotic communities and environmental parameters

**Fig. 6:**
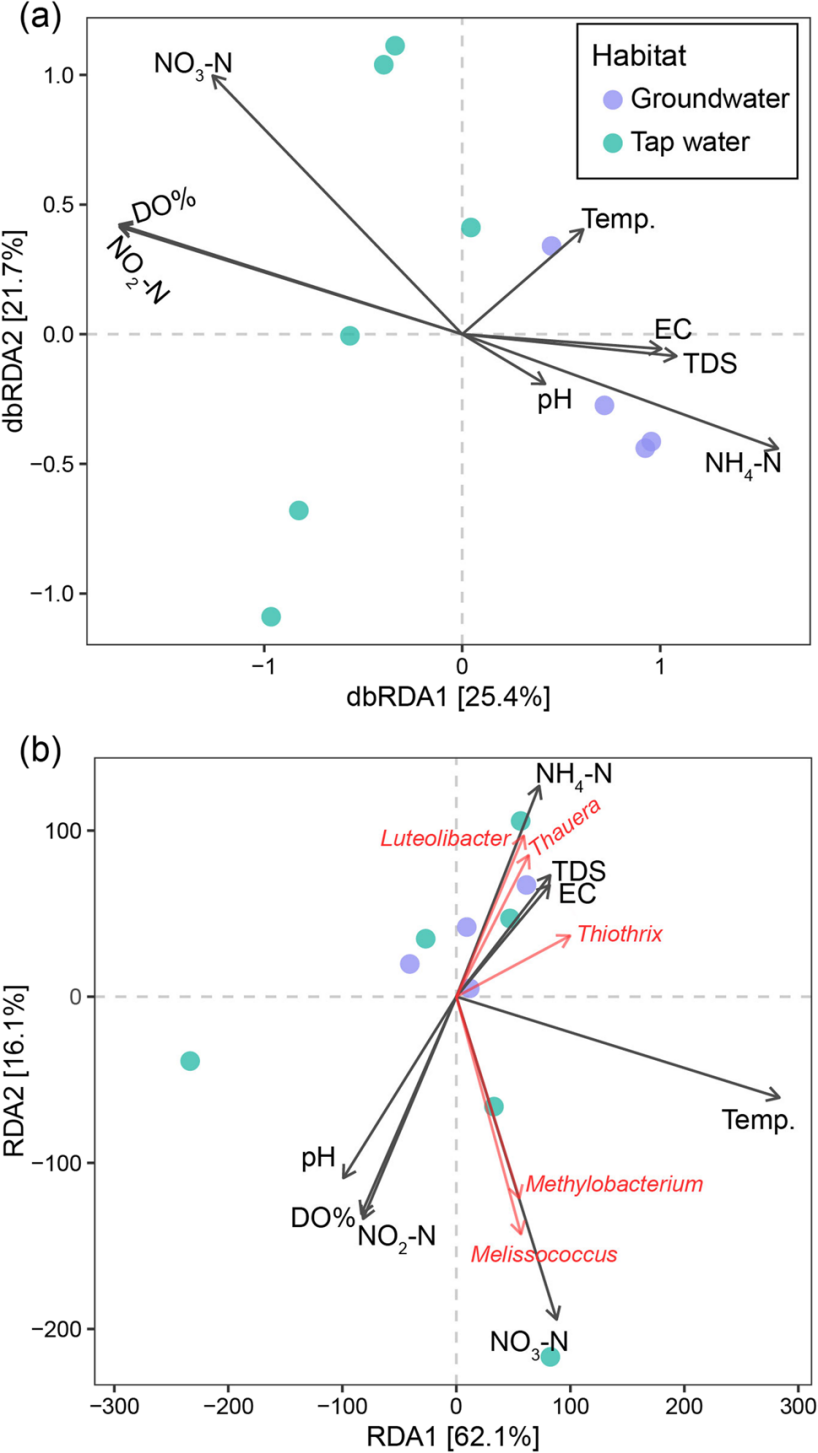
Distance-based redundancy analysis (dbRDA) plot showing (**a**) the relationships between prokaryotic community composition, environmental parameters in different habitats and (**b**) the associations between specific prokaryotic taxa and environmental parameters. Environmental factors, including pH, temperature (temp.), dissolved oxygen (DO%), electrical conductivity (EC), total dissolved solids (TDS), and nitrogen species (NH_4_-N, NO_2_-N, NO_3_-N), are shown as vectors, illustrating their influence on community structure and taxa distribution

The dbRDA plot (Fig. 6a) illustrates the distinct differentiation of microbial communities between groundwater and tap water, with dbRDA1 explaining 25.4% of the variation and dbRDA2 accounting for 21.7%. The clustering of samples reflects unique community structures shaped by habitat-specific environmental factors. In tap water, key environmental drivers such as DO%, NO₂-N, and NO₃-N were identified as significant influencers of microbial communities. Conversely, groundwater microbial compositions were primarily shaped by NH₄-N and TDS.

The RDA plot (Fig. 6b) further illustrates the significant role of environmental variables—such as pH, temperature, dissolved oxygen saturation (DO%), electrical conductivity (EC), total dissolved solids (TDS), and nitrogen species (NH₄-N, NO₂-N, NO₃-N)—in shaping the distribution of microbial taxa. The first two axes, RDA1 and RDA2, accounted for 62.1% and 16.1% of the observed variation, respectively. Notably, NO_3_-N exhibited a positive correlation with genera such as *Melissococcus* and *Methylobacterium*, suggesting their preference for nitrogen-enriched environments typically associated with anthropogenic activities and water treatment processes. On the other hand, NH₄-N was strongly correlated with *Luteolibacter* and *Thauera*, suggesting their adaptability to ammonium-rich environments, such as organic-rich groundwater systems.

### Assembly mechanisms of prokaryotic communities in aquatic environments

**Fig. 7:**
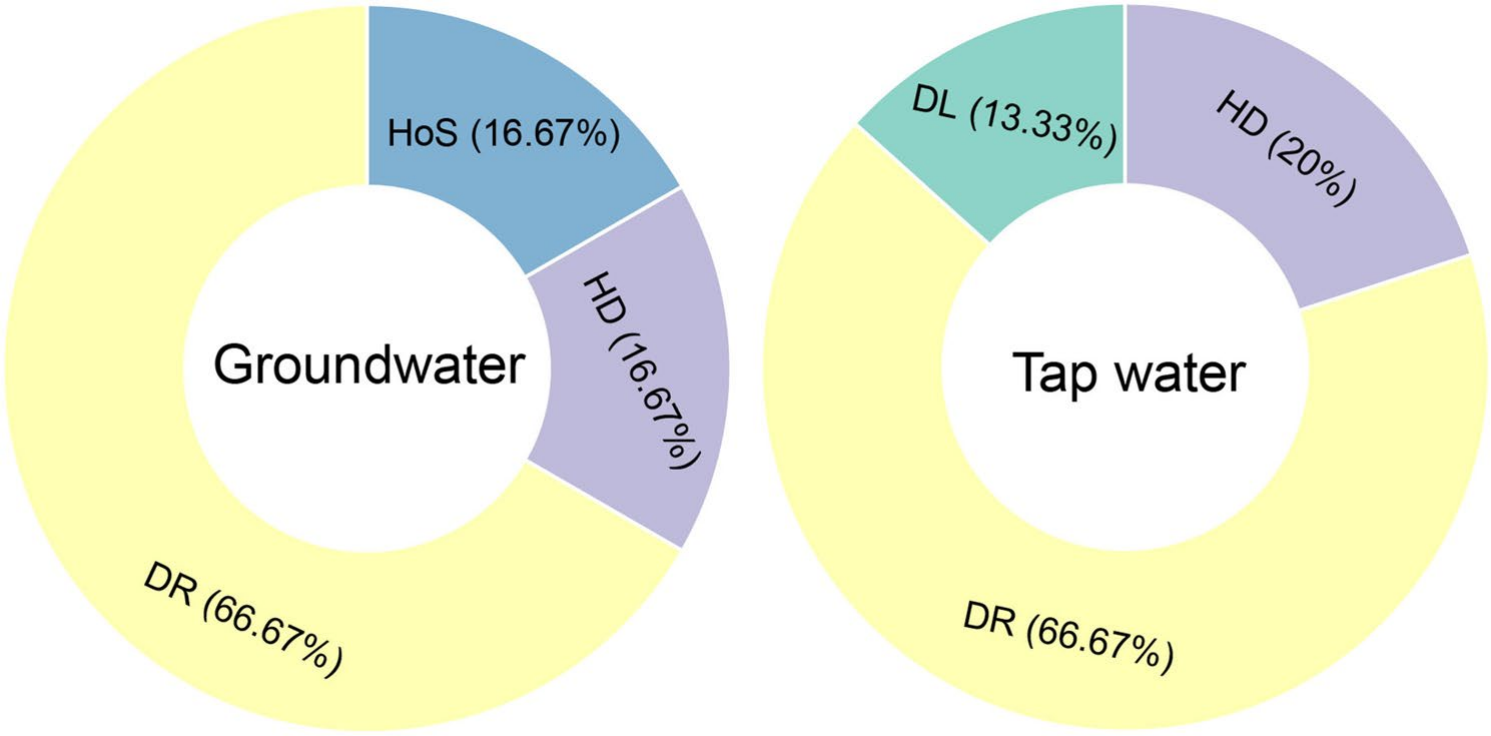
Proportions of ecological processes shaping prokaryotic community assembly in tap water and groundwater. The processes include DL (Dispersal Limitation), DR (Drift), HD (Homogenizing Dispersal), HeS (Heterogeneous Selection), and HoS (Homogenizing Selection)

The ecological processes governing prokaryotic community assembly were examined (Fig. 7). Distinct characteristics were observed in the processes shaping microbial communities in these two aquatic environments. In tap water, stochastic processes dominated, with DR accounting for 66.67% of the community assembly with HD contributed 20%, suggesting moderate microbial exchange and mixing, while DL played a smaller role at 13.33%. Notably, HeS and HoS were absent, indicating minimal deterministic influences in tap water. Similarly, in groundwater, DR was also the predominant process, contributing 66.67% to community assembly. HoS and HD each accounted for 16.67%, reflecting the effects of selective pressures and microbial mixing. These results underscore the differential influence of stochastic and deterministic forces in structuring microbial communities across groundwater and tap water systems (Fig. 7).

## Discussion

### Structural patterns and taxonomic profiling of prokaryotic communities in water systems

Ordination (NMDS) with confirmatory statistics (ANOSIM/Adonis) shows clear β-divergence between groundwater and tap water, reflecting distinct ecological filters imposed by aquifer conditions versus treatment and distribution. Similar source-to-tap stratification—where treatment reshapes communities but Proteobacteria remain dominant—has been reported in recent drinking-water investigations and in our prior work [1, 3, 10, 31]. Phylum-level rebalancing mirrors these filters: higher Firmicutes and Verrucomicrobiota in tap water are consistent with selection in oligotrophic, disinfected networks and biofilms, whereas Actinobacteriota and Bacteroidota (formerly Bacteroidetes) are relatively enriched in groundwater, matching subsurface nutrient/redox regimes [32, 33] At the class level, greater Betaproteobacteria in groundwater and higher Alphaproteobacteria/Bacilli in tap water align with differences in organic carbon availability, oxidized nitrogen, and residual disinfectant exposure typical of each habitat [34, 35]. These findings highlight the influence of environmental and treatment-related factors on microbial community structures, with notable differences in the distribution of key taxa between groundwater and tap water systems. Such insights are critical for understanding microbial ecology and its implications for water quality and safety.

Genus-level patterns further underscore functional niches shaped by these pressures. *Methylobacterium*—enriched in tap water—frequently persists in distribution-system biofilms and is recognized as an emerging opportunistic premise-plumbing pathogen [36]. By contrast, groundwater enrichment of *Thauera* and *Malikia* is consistent with denitrification-linked niches in subsurface environments [37]. Additional detections such as *Cutibacterium* in tap water (device-associated opportunist) illustrate that engineered systems can harbor low-abundance taxa with clinical relevance [38].

### Potential pathogens in aquatic systems

Our dataset revealed 15 potential human pathogens, with *Streptococcus salivarius* (ASV72, ASV165) most abundant and largely traced to drinking water (Fig. 5). Although typically an oral commensal, *S. salivarius* is a documented opportunist—including endocarditis, meningitis, and septicemia—particularly in compromised hosts [39]. *Stenotrophomonas maltophilia* appeared across multiple ASVs and at high relative abundance in drinking water, underscoring traits that favor persistence in engineered systems—biofilm formation and broad intrinsic/driven resistance (efflux pumps, L1/L2 β-lactamases, integron-mediated acquisition). Its detection post-treatment suggests either inadequate residuals or biofilm reservoirs within distribution networks [40].

Other opportunists—*Proteus vulgaris* (ASV1950), *Moraxella osloensis* (ASV155), *Acinetobacter baumannii* (ASV668), and *A. johnsonii* (ASV788)—were predominantly associated with drinking water, consistent with their known roles in healthcare-associated infections and resistance potential; their presence in treated supplies highlights the need for targeted monitoring and control of biofilms and residual disinfectants [41, 42]. Habitat specificity was also evident: *Ralstonia pickettii* (ASV1458) occurred exclusively in drinking water, aligning with its oligotrophic lifestyle and resilience to common disinfection methods, whereas *Prevotella melaninogenica* (ASV1560) predominated in groundwater (75.25%), a species associated with mucosal infections but also recovered from environmental waters [43, 44].

### Association between prokaryotic communities and environmental parameters

The dbRDA plot (Fig. 6a) illustrates the distinct differentiation of microbial communities between groundwater and tap water, with dbRDA1 explaining 25.4% of the variation and dbRDA2 accounting for 21.7%. The clustering of samples reflects unique community structures shaped by habitat-specific environmental factors. In tap water, key environmental drivers such as DO%, NO₂-N, and NO₃-N were identified as significant influencers of microbial communities. Conversely, groundwater microbial compositions were primarily shaped by NH₄-N and TDS. These findings are consistent with earlier research emphasizing the importance of DO, temperature, and nutrient concentrations in shaping microbial community composition in aquatic environments [45, 46].

The RDA plot (Fig. 6b) further illustrates the significant role of environmental variables—such as pH, temperature, dissolved oxygen saturation (DO%), electrical conductivity (EC), total dissolved solids (TDS), and nitrogen species (NH₄-N, NO₂-N, NO₃-N)—in shaping the distribution of microbial taxa. The first two axes, RDA1 and RDA2, accounted for 62.1% and 16.1% of the observed variation, respectively. Notably, NO_3_-N exhibited a positive correlation with genera such as *Melissococcus* and *Methylobacterium*, suggesting their preference for nitrogen-enriched environments typically associated with anthropogenic activities and water treatment processes. On the other hand, NH₄-N was strongly correlated with *Luteolibacter* and *Thauera*, suggesting their adaptability to ammonium-rich environments, such as organic-rich groundwater systems. These findings provide valuable insights into the ecological processes influencing microbial communities in aquatic systems. The identification of *Methylobacterium* and *Thauera* as key taxa linked to nutrient gradients underscores their potential as bioindicators for monitoring nutrient pollution and assessing water treatment efficiency. Effective management of nutrient levels, particularly nitrate and ammonium, is critical to mitigating microbial shifts that may compromise water quality or elevate the risks associated with opportunistic pathogens. These results emphasize the importance of integrated water resource management strategies to protect both human health and ecosystem stability.

### Assembly mechanisms of prokaryotic communities in aquatic environments

Null-model partitioning indicates drift (DR) dominated assembly in both habitats (tap and groundwater), with homogenizing dispersal (HD) contributing secondarily; only groundwater shows an added homogeneous selection (HoS) signal. Microbial community assembly in simulated cement and tap-water rainwater storage systems (SWSSs) appeared to be primarily governed by deterministic processes, which may be attributed to elevated pH levels and limited nutrient conditions in these environments [47]. In contrast, PVC and stainless-steel rainwater SWSSs were mainly influenced by stochastic processes, consistent with the high stochastic ratios (> 90%) reported for drinking water systems originating from conventional treatment plants [3]. Ecological drift, characterized by random processes like birth, death, reproduction, and extinction, has been identified as a critical factor in shaping community structures, particularly for smaller local populations [20]. Rare taxa, in particular, are susceptible to DR, with minor reductions in abundance potentially leading to species extinction, as seen with local chlorination effects [48]. The increased influence of DR along treatment disturbances further underscores its significance in microbial dynamics [3]. Unlike surface water, microbial communities in groundwater appear to experience a greater influence of homogenizing selection, a deterministic process linked to environmental factors [10, 21]. These insights are critical for understanding the complex interplay of stochastic and deterministic forces in microbial ecology and their implications for water quality management.

### Limitations

This study was based on a relatively limited number of samples collected during a single season and from one geographic region, which may reduce the broader applicability of the findings and influence the interpretation of microbial community assembly patterns. In addition, 16 S rRNA amplicon data cannot confirm microbial viability, virulence, or antibiotic resistance. Moreover, temporal variability was not assessed; therefore, future work should include multi-season sampling across a broader range of aquatic habitats.

## Conclusion

This study highlights clear differences in the microbial ecology of groundwater and tap-water systems in Egypt, shaped by habitat-specific environmental conditions. Using environmental DNA metabarcoding, we observed distinct community structures between the two water sources, with DO%, NO₂-N, and NO₃-N emerging as key correlates of tap-water communities, while NH₄-N and TDS were more strongly associated with groundwater communities. Although Proteobacteria dominated both habitats, the enrichment of taxa such as *Methylobacterium* in tap water and *Thauera* and *Legionella* in groundwater likely reflects the combined effects of natural hydrochemical conditions, treatment processes, and distribution-system dynamics.

Notably, signatures of several opportunistic and potentially pathogenic taxa—including *Streptococcus salivarius*, *Stenotrophomonas maltophilia*, *Acinetobacter baumannii*, and *Ralstonia pickettii*—were detected, particularly in tap-water samples. Because 16 S rRNA–based detection does not confirm viability, infectivity, or antimicrobial resistance, these findings should be interpreted cautiously. Nevertheless, their occurrence is consistent with possible post-treatment influences such as biofilm-associated persistence or distribution-related recontamination, underscoring the need for strengthened microbiological surveillance and distribution-system management. Future work incorporating larger, multi-season sampling and targeted viability-aware methods will be essential to support evidence-based interventions to protect public health, especially for vulnerable populations.

We recommend using this study’s pathogen findings to support a WHO-aligned review and update of national drinking-water standards. In addition, apply current guidance on opportunistic pathogens in distribution systems to define targets for disinfectant residual maintenance, biofilm control, and asset hygiene. To strengthen the evidence base, extend the work to multi-season, multi-site sampling across the full source-to-tap continuum (source water, treatment, distribution, and premise plumbing). Meanwhile, incorporate targeted culture and qPCR for the identified pathogens into routine monitoring and link results to predefined corrective actions. Finally, align pipe rehabilitation, flushing, storage management, and residual maintenance with relevant standards and utility best practices.

## Data Availability

The 16 S rRNA gene sequencing data generated in this study is publicly available in the NCBI Sequence Read Archive (SRA) under the BioProject accession number PRJNA1209216.

## Abbreviations

ASVs: Amplicon sequence variants
βNRI: Beta Net Relatedness Index
dbRDA: Distance-based redundancy analysis
DL: Dispersal limitation
DR: Drift
HD: Homogenizing dispersal
HeS: Heterogeneous selection
HoS: Homogeneous selection
nMDS: Non-metric multidimensional scaling
PVC: Polyvinyl chloride
RC: Raup–Crick index
RDP: Ribosomal Database Project
SWSSs: Simulated cement and tap-water rainwater storage systems
WHO: World Health Organization
